# Supplementary material for: From Hen Nutrition to Baking: Effects of Pomegranate Seed and Linseed Oils on Egg White Foam Stability and Sponge Cake Quality
Source: Foods. 2025 Apr 20;14(8):1417. doi: 10.3390/foods14081417 (PMC12026570; doi:10.3390/foods14081417)
Supplement: Supplementary file 1 [file foods-14-01417-s001.zip › foods-3545182-supplementary.pdf]

From Hen Nutrition to Baking: Effects of Pomegranate Seed and Linseed Oils on Egg White Foam Stability and Sponge Cake Quality

Supplementary materials

Marcin Lukasiewicz, Maja Dymińska-Czyż , Beata Szymczyk, Magdalena Franczyk-Żarów, Renata Kostogrys, Adam Florkiewicz , Paweł Ptaszek, Gabriela Zięć and Agnieszka Filipiak-Florkiewicz

Table S1. Composition of lying hens feed - Experiment 1

Item	Control group (A) g/kg	Experimental groups		
		B	C	D
Wheat	255	255	255	255
Maize	345	345	345	345
Soybean meal (460 g/kg crude protein)	217	217	217	217
Dried grass	30	30	30	30
Limestone	85	85	85	85
Dicalcium phosphate	17	17	17	17
Sodium chloride	3	3	3	3
Vitamin and mineral premix[1]	5	5	5	5
DL-Methionine	1	1	1	1
L-Lysine hydrochloride	2	2	2	2
Rapeseed oil	25	20	15	10
Linseed oil	15	15	15	15
Pomegranate seed oil	0	5	10	15
**Calculated nutrient content (g/kg)**				
Metabolizable energy (MJ/kg)[2]	11.55			
Crude protein	169.2			
Crude fibre	3.7			
Methionine + Cystine	7.4			
Lysine	8.4			
Sodium	1.7			
Calcium	36.5			

Chloride	1.7
Available phosphorous	6.8

Table S2. Composition of lying hens feed - Experiment 2

Item	Control group (A) g/kg	Experimental groups		
		B	C	D
Wheat	255	255	255	255
Maize	345	345	345	345
Soybean meal (460 g/kg crude protein)	217	217	217	217
Dried grass	30	30	30	30
Limestone	85	85	85	85
Dicalcium phosphate	17	17	17	17
Sodium chloride	3	3	3	3
Vitamin and mineral premix[1]	5	5	5	5
DL-Methionine	1	1	1	1
L-Lysine hydrochloride	2	2	2	2
Rapeseed oil	40	35	30	25
Pomegranate seed oil	0	5	10	15
**Calculated nutrient content (g/kg)**				
Metabolizable energy (MJ/kg)[2]	11.55			
Crude protein	169.2			
Crude fibre	3.7			
Methionine + Cystine	7.4			
Lysine	8.4			
Sodium	1.7			
Calcium	36.5			
Chloride	1.7			
Available phosphorous	6.8			

[1]The premix provided per 1 kg of diet: vitamin A—10.000 IU (retinol); vitamin D3—2000 IU (cholecalciferol); vitamin E—20 IU (dl-alpha-tocopherol); vitamin K3—1.5 mg (menadione); vitamin B1—1 mg (thiamine); vitamin B2—4 mg (riboflavin); vitamin B6—1.5 mg (pyridoxine); vitamin B12—0.02 mg (cyanocobalamin); biotin—0.05 mg Ca-pantothenate—8.7 mg; niacin—20 mg; folic acid—0.8 mg; choline chloride—200 mg; manganese—85 mg; zinc—60 mg; iron—45 mg; copper—8 mg; iodine—1 mg; selenium—0.25 mg; [2]Calculated according to Janssen, W.M.M.A. European Table of Energy Values for

Poultry Feedstuffs, 3rd ed.; Subcommittee Energy of the Working Group nr. 2 Nutrition of the European Federation of Branches of the World's Poultry Science Association: Beekbergen, The Netherlands, 1989; ISBN 90-71463-00-0. [Google Scholar].

Table S3. Fatty acid profile of oils used in all experiments, %

Fatty acids	RO	LO	PSO
C 12:0	-	-	0.03
C 14:0	0.20	0.05	0.06
C 16:0	4.40	6.5	6.35
C 17:0	0.04	0.05	0.09
C 18:0	2.0	3.5	3.36
C 18:1	60.5	18.1	16.23
C 18:2 n-6	20.1	15.0	13.74
C 18:3 n-3	11.8	56.78	0.41
C 20:0	0.66	0.02	0.70
C 20:1	-	-	0.96
C 24:0	0.30	-	0.20
CLnA	-	-	58.83
SFA	7.60	10.12	10.77
MUFA	60.50	18.10	15.59
PUFA	31.90	71.78	73.0

Table S4. The significance of the differences; p-values – Experiment 1 (ANOVA)

**Parameter**	
Hardness	0.5163
Cake weight	0.0560
Volume of the cake	**0.0182**
Dry matter	0.1281
Protein	**0.0312**
Fat	**0.0110**
Ash	0.4115
C14:0 tetradecanoic (myristic acid)	**0.0211**

C15:0 pentadecanoic (pentadecylic acid)	0.1532
C16:0 hexadecanoic (palmitic acid)	**0.0030**
C17:0 heptadecanoic (margaric acid)	0.0210
C18:0 octadecanoic (stearic acid)	0.5422
C20:0 eicosanoic acid (arachidic acid)	0.3317
C22:0 docosanoic (behenic acid)	**0.0001**
C14:1 9-tetradecenoic (myristoleic acid)	**0.0000**
C16:1 *trans*-3-hexadecenoic	**0.0331**
C16:1 9-cis-Hexadecenoic (palimitoleic acid)	**0.0010**
C17:1 10-heptadecenoic acid	0.4871
C18:1 *cis*-9-Octadecenoic (oleic acid)	0.5595
C22:1 (13Z)-docos-13-enoic (erucic acid)	**0.0011**
C16:2 Hexadecadienoic acid	**0.0201**
C18:2 *n*−6 *cis,cis*-9,12-octadecadienoic (linoleic acid)	**0.0051**
C18:2 Conjugated linoleic acids - CLA	**0.0110**
C18:3 *n*-3 *cis,cis,cis*-9,12,15-octadecatrienoic (α-linolenic acid)	**0.0037**
C18:3 Conjugated linolenic acid - CLnA	**0.0056**
C20:2 eicosadienoic	**0.1897**
C20:3 *n*-6 *cis,cis,cis*-8,11,14-eicosatrienoic dihomo-γ-linolenic acid	**0.0000**
C20:4 *n*-6 5,8,11,14-*all-cis*-eicosatetraenoic (arachidonic acid)	**0.0212**
C22:6 *n*-3 docosahexaenoic - DHA (cervonic acid)	**0.0011**
Other C18:2, C18:3, CLA	**0.0000**
Saturated fatty acids - SFA (%)	0.3887
Monounsaturated fatty acids - MUFA (%)	**0.0411**
Polyunsaturated fatty acids - PUFA (%)	**0.0028**
Foaming capacity	0.6321
Stability of foams	**0.0349**
Foam index	0.2433
Percentage of gas in the foam	0.4832
Foam density	0.5517

Table S5. The significance of the differences; p-values – Experiment 2

Parameter	
Hardness	0.4138
Cake weight	0.7891
Volume of the cake	0.7713
dry matter	**0.0204**
protein	**0.0154**
fat	**0.0004**
ash	0.6811
C14:0 tetradecanoic (myristic acid)	**0.0011**
C15:0 pentadecanoic (pentadecylic acid)	**0.0210**
C16:0 hexadecanoic (palmitic acid)	**0.0000**
C17:0 heptadecanoic (margaric acid)	**0.0142**
C18:0 octadecanoic (stearic acid)	**0.0051**
C20:0 eicosanoic acid (arachidic acid)	0.5331
C22:0 docosanoic (behenic acid)	**0.0003**
C14:1 9-tetradecenoic (myristoleic acid)	**0.0412**
C16:1 *trans*-3-hexadecenoic	**0.0021**
C16:1 9-cis-Hexadecenoic (palimitoleic acid)	**0.0033**
C17:1 10-heptadecenoic acid	0.4170
C18:1 *cis*-9-Octadecenoic (oleic acid)	**0.0028**
C22:1 (13Z)-docos-13-enoic (erucic acid)	**0.0100**
C16:2 Hexadecadienoic acid	**0.0031**
C18:2 *n*−6 *cis,cis*-9,12-octadecadienoic (linoleic acid)	**0.0214**
C18:2 Conjugated linoleic acids - CLA	**0.0000**
C18:3 *n*-3 *cis,cis,cis*-9,12,15-octadecatrienoic (α-linolenic acid)	**0.0044**
C18:3 Conjugated linolenic acid - CLnA	**0.0121**
C20:2 eicosadienoic	0.3100
C20:3 *n*-6 *cis,cis,cis*-8,11,14-eicosatrienoic dihomo-γ-linolenic acid	**0.0120**
C20:4 *n*-6 5,8,11,14-*all-cis*-eicosatetraenoic (arachidonic acid)	**0.0007**
C22:6 *n*-3 docosahexaenoic - DHA (cervonic acid)	**0.0327**
Other C18:2, C18:3, CLA	**0.0000**
Saturated fatty acids - SFA (%)	0.5277

Monounsaturated fatty acids - MUFA (%)	**0.0021**
Polyunsaturated fatty acids - PUFA (%)	**0.0180**
Foaming capacity	**0.0210**
Stability of foams	**0.0001**
Foam index	**0.0319**
Percentage of gas in the foam	**0.0021**
Foam density	**0.0000**
